# Pollination mutualism between *Alocasia macrorrhizos* (Araceae) and two taxonomically undescribed *Colocasiomyia* species (Diptera: Drosophilidae) in Sabah, Borneo

**DOI:** 10.1111/j.1438-8677.2011.00541.x

**Published:** 2012-01-30

**Authors:** K Takenaka Takano, R Repin, M B Mohamed, M J Toda

**Affiliations:** 1 Graduate School of Environmental Earth Science, Hokkaido UniversitySapporo, Japan; 2 Sabah Parks, Kota KinabaluSabah, Malaysia; 3 Institute for Tropical Biology and Conservation, Universiti Malaysia SabahKota Kinabalu, Sabah, Malaysia; 4 Institute of Low Temperature Science, Hokkaido UniversitySapporo, Japan

**Keywords:** *Colocasiomyia* sp.1 aff. *sulawesiana*, *Colocasiomyia* sp.2 aff. *sulawesiana*, Kota Kinabalu, life history, pistilicolous species, pollination experiment

## Abstract

Two taxonomically undescribed *Colocasiomyia* species were discovered from inflorescences of *Alocasia macrorrhizos* in Kota Kinabalu City, Sabah, Borneo, Malaysia. The aims of this study were to investigate the reproductive ecology of the flies and the plant, ascertain the importance of the flies as pollinators and examine the intimate association between flowering events and life history of the flies. We conducted sampling, observations and field pollination experiments. The flies were attracted by the odour of female-phase inflorescences in the early morning on the first day of anthesis. They fed, mated and oviposited in the inflorescences for 1 day. On the second day, the flies, covered with pollen grains, left the male-phase inflorescences for the next female-phase inflorescences. The immature forms of both fly species hatched, developed and pupated within the infructescences without damaging the fruits, and developed adults emerged when the mature infructescences dehisced. The flowering events and fly behaviours were well synchronized. In field pollination experiments, inflorescences bagged with a fine mesh (insect exclusion) produced almost no fruits, whereas those bagged with a coarse mesh (bee exclusion) produced as many fruits as the open-pollinated controls. These results indicate that these flies are the most efficient and specialised pollinators for their host, *A. macrorrhizos*. These flies, in return, depend on *A. macrorrhizos* for food and habitat through most of their life cycle. This study provides a deeper insight into the less recognised, highly intimate pollination mutualism between Araceae plants and *Colocasiomyia* flies.

## Introduction

The Araceae have a unique and characteristic inflorescence made up of a spadix and a spathe. All the inflorescence morphologies observed in the family Araceae can be seen as variations around this same theme (Bown 2000). In spite of their constant inflorescence design, Araceae have developed a great diversity of pollination systems (Vogel 2000; Gibernau 2003), partly because of the evolution of unisexual flowers that has allowed the secondary development of sterile flowers, as well as floral function specialisation such as barrier, odour emission, thermogenesis and food-reward (Mayo *et al.* 1997; Gibernau 2003). Development of an enclosing spathe (*i.e.*, a floral chamber) with secondary appearance of a constriction allows the capture of insects in contact with the flowers (Lack & Diaz 2001; Gibernau 2003). In species with a floral chamber, once pollinators have been attracted during the female phase, they are then kept within the floral chamber by trap mechanisms or rewards (food, mating partners, shelter from light, *etc*.) until the end of anthesis (*i.e.*, pollen release) hours or a few days later (Lack & Diaz 2001; Gibernau 2003).

The flowers of several plant families serve as breeding places for pollinator insects. In many cases, saprophagous flies or beetles pollinate the host plants, and the decaying floral parts (*e.g.*, corollas or male flowers) in turn serve as food for these insects (Sakai 2002). In some Araceae, their inflorescences also serve as reproductive sites whereas others mimic the laying site (*i.e.*, faeces, fungi and dead animals) of the pollinator flies (Gibernau 2003; Seymour *et al.* 2003a).

Members of the genus *Colocasiomyia* de Meijere, 1914 (Diptera: Drosophilidae), which currently consists of about 70 species, are found only on flowers of Araceae, Arecaceae and Magnoliaceae (Sultana *et al.* 2006; Takenaka 2006). In the case of some species, oviposition and larval development take place on host inflorescences (Carson & Okada 1980; Toda & Okada 1983) and the flies serve as major species-specific pollinators (Yafuso 1993; Mori & Okada 2001; Takenaka *et al.* 2006). The close association between certain species groups within the genus *Colocasiomyia* and certain host taxa suggests that these insects have had long evolutionary relationships with their host plants (Sultana *et al.* 2006).

Sharing of a single aroid inflorescence by a pair of fly species with partial niche separation is a widely observed ecological trait of *Colocasiomyia* flies; a pistilicolous (*pistil*: female flower, -*colous*: a suffix from the Latin word meaning *inhabiting*) species uses the female inflorescence for oviposition and larval development, whereas a stamenicolous (*i.e*., inhabiting in male flowers) species mostly uses the male inflorescence (Carson & Okada 1980). Different pairs of fly species have been found on different aroid host species or species from different geographic regions (Okada 1975, 1980, 1986; Carson & Okada 1980; Honda-Yafuso 1983; Toda & Okada 1983; Okada & Yafuso 1989; Yafuso & Okada 1990). Further, additional *Colocasiomyia* species are continuously being discovered (Sultana *et al.* 2002; Sultana *et al.* 2006; Takenaka 2006; Takenaka *et al.* 2006; Toda & Lakim 2011) and show different patterns of species coexistence: some monopolise an inflorescence whereas others coexist with up to seven other species (Takenaka 2006; Takenaka *et al.* 2006; Toda & Lakim 2011).

There is high host specificity within the *Colocasiomyia cristata* species group; the flies reproduce exclusively on inflorescences of the genera *Colocasia*, *Alocasia* and *Steudnera* (Araceae) and each fly species is usually associated with just one or two host plant species (Carson & Okada 1980; Miyake & Yafuso 2005; Takenaka 2006; Toda & Lakim 2011). *Alocasia macrorrhizos* (L.) G. Don is the most widely distributed species of *Alocasia* and is visited by different *Colocasiomyia* species in different regions (Okada & Yafuso 1989; Yafuso & Okada 1990; Sultana *et al.* 2006; Takenaka 2006; Toda & Lakim 2011). In 1999, two previously undescribed *Colocasiomyia* species were found to coexist in the inflorescences of *A. macrorrhizos* in Sabah, Malaysian Borneo (Toda & Lakim 2011). In 2004, we revisited the same locality with the aims to determine (i) the flowering ecology of the host plant, (ii) the association between flowering events and fly behaviour, (iii) the importance of the flies as pollinators in comparison with other flower visitors, and (iv) the reproductive habits of the flies. Finally, we characterise the unique pollination mutualism between Araceae plants and *Colocasiomyia* flies.

## Material and Methods

### Host plant

**Figure 1:**
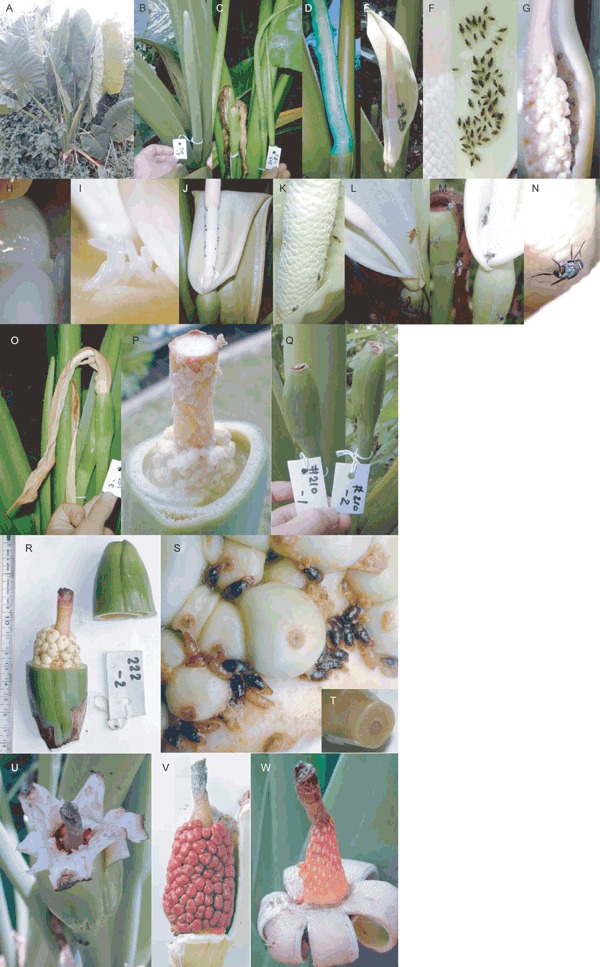
Flowering and fruiting sequences of *Alocasia macrorrhizos* associated with *Colocasiomyia* flies and other flower visitors in Kota Kinabalu. A: A ramet of *A. macrorrhizos*. B: A bud of a synflorescence (paired inflorescences). C: Pairs of buds (Stage I) and developing infructescences (Stages IV and V). D: *Colocasiomyia* flies just arrived on a female-phase inflorescence (Stage II) in the morning twilight. E, F: *Colocasiomyia* flies gathered in the upper and (G) the lower parts (dissected spathe tube) of the spathe chamber. Eggs of (H) *Colocasiomyia* sp.1 aff. *sulawesiana* and (I) *Colocasiomyia* sp.2 aff. *sulawesiana* laid in the spaces between pistillate flowers. J–L: An inflorescence releasing pollen (Stage III) early in the morning. J, K: *Colocasiomyia* flies crawling up the spadix being dusted with pollen grains and (L) honeybees collecting pollen. M: Stingless bees collecting pollen deposited on the upper spathe chamber after pollen release (Stages III and IV). N: ?*Atherigona* species on an inflorescence (Stage IV). O: Young infructescence at Stage IV. P: A dissected infructescence filled with secretion in which *Colocasiomyia* larvae developed. Q: Developing infructescences at Stage V. R, S: A dissected infructescence with *Colocasiomyia* pupae in the spaces between fruits and the spathe tube. T: The top of an infructescence tightly enclosed with the spindle and the spathe tube (dissected). U: An infructescence starting to dehisce at Stage VI. V: A dissected infructescence with red mature fruits and *Colocasiomyia* puparia. W: Remains of an infructescence several days after dehiscence.

**Figure 2:**
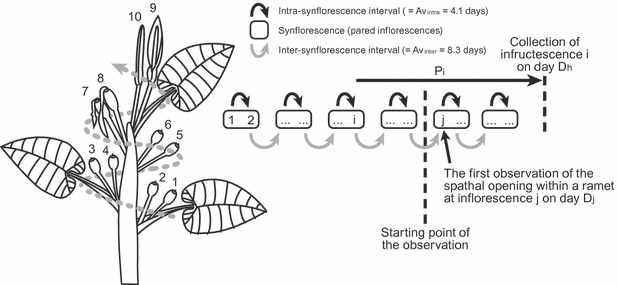
Schematic of the flowering sequence within a ramet (dashed arrow, left) and estimation of the period from the day of spathe opening to the day of collection of the infructescence. In this example, equation (1) becomes P_i_ = (D_h_ − D_j_) + (4.1 × 1) + (8.3 × 2) = (D_h_ − D_j_) + 20.7. P_i_ was calculated in this manner for each collected infructescence to estimate the age of the *Colocasiomyia* immatures inside.

**Figure 3:**
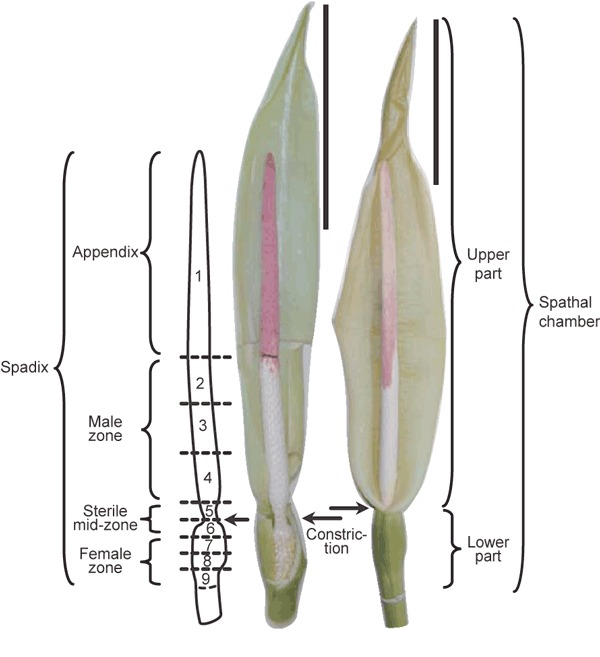
Schematic of a spadix (left), an inflorescence with the lower spathe removed (centre) and an intact inflorescence (right) of *Alocasia macrorrhizos*. The numbered sections correspond to those in Table S2. Scale bar = 10 cm. Interestingly, the appendix of some inflorescences became pinkish at the end of flowering.

*Alocasia macrorrhizos* is a perennial herb with a thick erect stem and is found along the edges and open gaps of forests, as well as along roadside across Indo-Malesia and Oceania (Fig. 1). Its natural origin is unknown but is likely to be within Southeast Asia. In some Pacific regions, it is traditionally cultivated as a starchy stem crop and has a long history of human use and dispersal (Hay & Wise 1991; Hay *et al.* 1995; Hay 1998). Synflorescences (paired inflorescences) bloom one by one as a pair per leaf and such pairs occur alongside each other in a continuous sequence (Fig. 2). From the bottom to the top, the spadix has a female zone, a sterile mid-zone, a male zone and a sterile appendix (Fig. 3). A cream-coloured spathe covers the spadix and forms a floral chamber, which constricts around the sterile mid-zone. The upper spathe and spadix begin to decay soon after pollen releasing phase (Fig. 1O), wither and then drop off (Fig. 1Q). An infructescence of an old and big plant usually contains more than 100 fruits or berries and each fruit contains 1–5 seeds.

Voucher specimens were deposited in the herbaria of the Institute for Tropical Biology and Conservation, Universiti Malaysia Sabah (specimen KT-303) and the headquarters of Kinabalu Park (specimens KT-303 and 305).

### Study site

Sampling, observations and experiments were conducted at a vacant lot in Kota Kinabalu City (5°58′32′′ N, 116°04′29′′ E, 15 m a.s.l.), Sabah, Borneo, Malaysia. Patches of *A. macrorrhizos* are common in open areas throughout the city and the plant blooms continuously. A landowner established the patch at our study site several years previously, and the patch is pruned for control approximately every 3 months.

### Ecological observation and sampling

We observed the inflorescences of 23 ramets and the insect visitors between 10:00 and 14:00 h daily from 14 July to 1 August 2004, and conducted continuous observations from 04:40 to 22:20 h from 22 to 25 July 2004. We defined the stages in the flowering and fruiting sequence as follows:

Stage I – emergence from the leaf sheath to just before opening of the spathe (Fig. 1B and C);

Stage II – pollen-receiving phase (*i.e.*, female phase) for a 1-day period, with open spathe and *Colocasiomyia* flies (Fig. 1E–G);

Stage III – pollen-releasing phase (*i.e*., male phase), during which the flies are covered in pollen (Fig. 1J and K);

Stage IV – early swelling of the infructescence and initial decay of the upper spathe and spadix, from just after the pollen-release phase (Fig. 1O);

Stage V – late swelling of the infructescence, from withering and fall of the upper spathe and spadix until dehiscence of the lower spathe (Fig. 1R); and

Stage VI – appearance of red mature fruits in the infructescence after dehiscence (Fig. 1U and V).

In total, 288 inflorescences and infructescences of the 23 ramets were marked and the flowering and fruiting sequence was followed for each.

#### Flower visitors

*Colocasiomyia* flies often gathered in the upper (Fig. 1E and F) and lower (Fig. 1G) parts, separately, of the spathe chamber (Fig. 3). We collected visiting insects from the upper part by direct aspiration. Thereafter, the entire inflorescence was covered with a plastic bag and detached from the plant. Insects remaining in the lower part were aspirated from inside the bag. The insects were initially preserved in Kahle’s fluid (distilled water, 95% ethanol, formaldehyde and glacial acetic acid in a 28:17:6:2 ratio) and later stored in 70% ethanol. *Colocasiomyia* flies were identified to the species level, and other insects were identified to genus, family or order level. The *Colocasiomyia* species composition and sex ratio in the upper and the lower parts of each inflorescence were compared using Fisher’s exact test with JMP 7 software (SAS Institute, Cary, NC, USA). Voucher specimens of all insect taxa have been kept for future reference.

### Immature stages of *Colocasiomyia* in the infructescences

We examined five inflorescences and 22 infructescences at different stages in the laboratory. The spadix was cut into nine sections (Fig. 3); the male and female zones were divided into three sections of equal length, and the sterile mid-zone was divided into two sections of equal length. The total number of immature individuals and adults, awaiting eclosion in puparia, was counted under a stereomicroscope for each section and species. The diagnostic morphological characteristics of immatures of the two taxonomically undescribed *Colocasiomyia* species and another diptera species are listed in Table S1. The developmental stages were identified as follows: egg, first-instar larva, second- and third-instar larva, pupa, puparium before eclosion and empty puparium after eclosion. The distribution data on eggs and first-instar larvae were summed for each *Colocasiomyia* species (Table S2) and then compared using the chi-square test with JMP 7 software.

#### Age estimation of *Colocasiomyia* immatures by infructescences

The *Colocasiomyia* flies laid eggs on an inflorescence in Stage II, which lasts for only 1 day. Therefore, we could estimate the age of the fly progeny that develop in the inflorescence as being equal to the period following Stage II; we recorded this period from the beginning of Stage II to the harvesting date of each infructescence.

For developing infructescences in Stages IV and V, we estimated the time since egg deposition by tracing back the infructescence sequence because the inflorescences bloom at regular intervals. We named the interval between the first and the second inflorescence within a synflorescence as the ‘intra-synflorescence interval’ and the interval between the second inflorescence of a preceding (*i.e*., older) synflorescence and the first inflorescence of the following (*i.e*., younger) synflorescence as the ‘inter-synflorescence interval’ (Fig. 2). Then, P_i_, which is defined as the number of days from the beginning of Stage II to the harvesting of infructescence i, is calculated as:


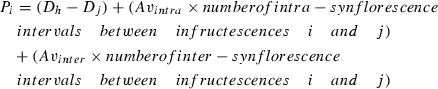
1

where infructescence j is the first inflorescence, within a ramet, that opened the spathe after we started the observation; D_j_ is the date of the beginning of Stage II of inflorescence j; D_h_ is the harvesting date of infructescence i; Av_intra_ is the average duration of the intra-synflorescence intervals; and Av_inter_ is the average duration of the inter-synflorescence intervals.

To investigate the time of *Colocasiomyia* adult emergence from the infructescences, we covered some infructescences before dehiscence with nylon stockings.

### Field pollination experiments

Three inflorescences for each of 13 ramets were treated to examine the importance of each insect group as pollinators: one inflorescence was tagged (open-pollinated control), another was bagged with a coarse mesh (2-mm grid, soap bag) to exclude large insect visitors (Fig. 1D) and the third was bagged with a fine-mesh nylon stocking to exclude *Colocasiomyia* and all other insect visitors. The treatments were performed from 14 to 26 July 2004 and the bags were removed after flowering. Usually, *Alocasia* fruits mature in 45–90 days. However, we collected the developing infructescences before maturation, on 12 August 2004, and preserved them in Kahle’s fluid, because of the schedule of the field trip.

The numbers of developing and undeveloped fruits were counted for each infructescence. We discriminated between the developing and undeveloped fruits by examining whether a fruit had one or more developing seeds, using a stereomicroscope. The fruit fertility rate was estimated by dividing the number of developing fruits by the total number of fruits (both developing and undeveloped) for each infructescence. We compared the fertility rates between the control treatment and the other treatments using the Wilcoxon matched pairs signed-ranks test using JMP 7.

## Results

### Flowering and fruiting sequence

The mean total number of inflorescences and infructescences per ramet was 12.5 (SD = 2.6, maximum = 18, n = 23). The mean durations of the stages in the flowering and fruiting sequence were 1.3 days in Stage II (SD = 0.4, n = 32), 1.0 day in Stage III (SD = 0.0, n = 29) and 5.0 days in Stage IV (SD = 1.0, n = 14). None of the flowering periods from Stage II (female phase) to Stage III (male phase) overlapped between inflorescences within a ramet, presumably to avoid geitonogamy. The mean intra-synflorescence interval (Av_intra_) was 4.1 days (SD = 0.7, n = 18) and the mean inter-synflorescence interval (Av_inter_) was 8.3 days (SD = 1.2, n = 16). Thus, equation (1) becomes


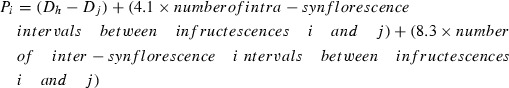
2

Using equation (2), we estimated the ages of the immatures of the *Colocasiomyia* flies within the harvested infructescences (Table S3).

### Flowering events and behaviours of the *Colocasiomyia* flies

Two fly species, *Colocasiomyia* sp.1 aff. *sulawesiana* and *Colocasiomyia* sp.2 aff. *sulawesiana* (hereafter abbreviated as Sp. 1 and Sp. 2, respectively) were the predominant visitors (Table S3). We often observed many pollen grains attached on bodies of collected flies of both Sp. 1 and Sp. 2 (Fig. 1K). Their behaviours corresponded well to the flowering events described below.

Early on the first morning, the spathe opened and presented a narrow slit, and the floral chamber emitted a strong odour. The *Colocasiomyia* flies were attracted to the spathe and then entered through the slit around sunrise, between 05:50 and 06:20 h (Fig. 1D). The flies remained in the chamber for 1 day to feed, mate (Fig. 1E–G) and deposit eggs between pistils or staminodes (Fig. 1H and I). Some flies often congregated inside the upper spathe chamber (Fig. 1E and F), whereas others swarmed to the lower part (Fig. 1G). The species composition of the collected flies differed significantly between the upper and the lower parts in all the inflorescences examined: Sp. 1 was more abundant in the lower part and Sp. 2 was in the upper part (Table S3). The sex ratio, however, was not significantly different between the upper and the lower parts for either species, except for Sp. 2 in one inflorescence (Table S3).

After the 1-day female phase (Stage II), the male phase (Stage III) began with pollen release before 04:40 h on the second morning. Pollen release continued till 06:00 h, when the constriction of the spathe (Fig. 3) began to tighten (Fig. 1J). *Colocasiomyia* flies escaped from the lower chamber as it closed by crawling up the spadix in a shower of pollen (Fig. 1J). The flies flew away, presumably to enter female-phase inflorescences on the nearby ramets.

These floral events occurred every morning, with the *Colocasiomyia* flies migrating from one inflorescence to another and staying overnight in each temporary habitat.

### Other visitors to *A. macrorrhizos* inflorescences

Many stingless bees –*Trigona* (*Tetragonula*) *fuscobalteata* Cameron, 1908 and *Trigona* (*Tetragonula*) *laeviceps* Smith, 1857 (Hymenoptera: Apidae) – collected pollen before sunrise and in the daytime (Fig. 1M). Many of them visited the inflorescences only after pollen release, when the female zone was no longer accessible due to spathe closure. They sometimes investigated female-phase inflorescences and rarely entered the lower part of the spathe chamber, where the pistils are located.

In the morning, between 04:40 and 07:00 h, honeybees (*Apis cerana* Fabricius, 1793; Hymenoptera: Apidae) also visited the inflorescences that were releasing pollen (Fig. 1L).

Adult flies, which were tentatively identified as *Atherigona* sp. (Diptera: Muscidae), repeatedly visited the inflorescences and young infructescences of Stages II–IV in the daytime (Table S3, Fig. 1N), but not during pollen release in the early morning. They walked around the spadices that had been covered with pollen grains for a couple of days after pollen release and sometimes moved to the lower part of female-phase inflorescences. They used the inflorescences as reproductive sites and, presumably, as feeding sites (Fig. 1N). Eggs were laid on the sterile mid-zone and male zone (Table S2). Second- and third-instar larvae of the species fed on decaying tissue of the male zone and appendices (Table S2). Several pupae were found from the appendices to the male zones (Table S2). Some larvae and pupae were collected together with the decaying appendices and reared on the appendices at ambient room temperature until they became identifiable adults.

Two adult females of *Neurochaeta mcalpinei* Woodley, 1982 (Diptera: Neurochaetidae) and one adult female of *Stenomicra* (*Podocera*) *australis* Malloch, 1927 (Diptera: Periscelididae) were collected (Table S3), but their behaviours were not observed. Parasitoid wasps (Hymenoptera) were found in two inflorescences (Table S3), and several individuals were found developing in *Colocasiomyia* puparia (Table S4). Earwigs belonging to the species *Chelisoches morio* (Fabricius, 1775) (Dermaptera: Chelisochidae), were often present at the bottom of the spathe chambers. One rove beetle (Coleoptera: Staphylinidae) and one collembolan were also collected (Table S3).

### Field pollination experiments

**Figure 4:**
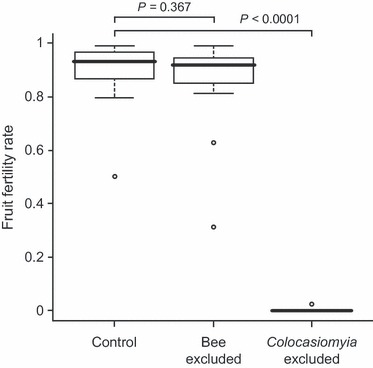
Comparison of the fruit fertility rate among the open-pollinated control, bagging with coarse mesh (bee exclusion) and bagging with fine mesh (*Colocasiomyia* exclusion) treatments (n = 13 for each treatment). The bold horizontal line shows the median fertility rate. The bottom and top of each box show the 25th and 75th percentiles (*i.e*., the first and the third quartiles), respectively. The vertical dashed lines are either the maximum value or 1.5 times the interquartile range of the data, whichever is smaller. Points more than 1.5 times the interquartile range above the third quartile and those more than 1.5 times the interquartile range below the first quartile are plotted individually. The P-values were obtained using a one-tailed Wilcoxon test in each comparison with the control.

The fruit fertility rates of the control, bee-excluded and *Colocasiomyia*-excluded inflorescences were 0.89 ± 0.13 (mean ± SD, n = 13), 0.85 ± 0.19 (n = 13) and 0.002 ± 0.007 (n = 13), respectively (Fig. 4). The inflorescences bagged with the coarse mesh were visited by *Colocasiomyia* flies but not larger insects, and produced as many fruits as the open-pollinated controls (Z = −5.5, P = 0.367, one-tailed Wilcoxon test for comparisons with the control) (Fig. 4). However, excluding *Colocasiomyia* and all or most of the other insects with the fine mesh reduced the seed production almost completely (Z = −45.5, P < 0.0001); only one of 13 inflorescences produced three fruits, although each inflorescence possessed more than 100 pistils.

### Immature stages of *Colocasiomyia* on the host inflorescences

Eggs and young larvae of Sp. 1 and Sp. 2 were found on the pistils of the female zone and the staminodes of the sterile mid-zone (Table S2; Fig. 1H and I). Their distributions in the infructescences were bimodal, with a larger peak in the lower part of the female zone and a smaller peak in the lower part of the sterile region (subtotal of Table S2), and significantly different between the species (χ^2^ = 407.5, df = 8, P < 0.0001; the numbers of individuals on Sections 4 and 5 were summed because of the small value).

Larvae of both *Colocasiomyia* species were found in the lower part of the male zone and throughout the female zone in Stage III. The larvae were subsequently found only in the female zone during Stage IV (Table S2), when the infructescence is bathed in its own secretion (Fig. 1P). The *Colocasiomyia* larvae developed in this secretion and seemed to feed on something from it (*e.g*., the secretion itself or bacteria or yeasts proliferating in the secretion). During the later part of Stage V, when the inside of the infructescences became drier (Fig. 1R), the larvae pupated in spaces between the fruits and the inner side of the spathe tube, especially where cavities formed around aborted fruits (Fig. 1S). The distributions of the larvae and puparia in the infructescences after Stage III were not significantly different between the species (data not shown).

We observed new adults of Sp. 1 and Sp. 2 emerging from a matured infructescence just after dehiscence. On the first day of dehiscence, the surrounding spathe tube began to open at the top (Fig. 1U), and a dozen new adults were observed in the stocking cover. On the second day, the spathe split further and more than 100 new adults emerged. Only three uneclosed pupae were found among the abundant empty puparia (Table S4; ramet 222 – inflorescence 1). The age of both the flies and the infructescence, estimated using equation (2), was approximately 74 days (Table S4; ramet 222 – inflorescence 1).

According to the estimated ages of the infructescences, the youngest infructescence in which empty puparia were found was 62.3 days after anthesis (ramet 224 – infructescence 3 at Stage VI, for both species) and the oldest one in which living puparia with a developed adult body were found was 89.0 days (ramet 232 – infructescence 1 at Stage V, for both species).

## Discussion

### Flowering events and behaviour of the *Colocasiomyia* flies

The flowering events and the behaviours of the *Colocasiomyia* flies were well synchronised. The *Colocasiomyia* flies pollinated their host in a sophisticated and effective manner, as has been reported for other pollination mutualisms between *Colocasiomyia* flies and their species-specific host plants in the Araceae (Carson & Okada 1980; Kramadibrata & Hambali 1983; Mori & Okada 2001; Takenaka 2006; Takenaka *et al.* 2006; see also Cleghorn 1913; Toda & Okada 1983; Yafuso 1993).

Ivancic *et al.* (2005) studied inflorescence heating (thermogenesis) of *A. macrorrhizos* in Vanuatu and reported that the average maximum temperature ± SEM of the appendix reached 43.9 ± 0.6 °C (n = 59 inflorescences; average ambient air temperature was 22.4 ± 0.5 °C) between 05:45 and 06:45 h on the first morning of anthesis. The function of the inflorescence thermogenesis in Araceae is generally agreed to be to volatilise odour compounds for pollinator attraction (Mayo *et al.* 1997). The time of flower visiting by the *Colocasiomyia* flies observed in the present study corresponded well to the time of the peak temperature reported in Ivancic *et al.* (2005). Seymour *et al.* (2003b) reported that floral heat of *Philodendron solimoesense* (Araceae) in French Guiana serves as a direct reward for a pollinating large scarab beetle, *Cyclocephala colasi* (Coleoptera: Scarabaeidae). It is unclear, however, whether floral heat of *A. macrorrhizos* serves as a direct reward for the *Colocasiomyia* flies.

### Other visitors to the inflorescences

The common visitors were stingless bees, but their role as pollinators (if any) seems to be minor, because they rarely accessed the pistils. Honeybees visited the inflorescences only when pollen was actively released in the early morning. Even if they were able to access the female-phase inflorescences, they are too large to enter the lower part of the spathe chamber and thus would not contribute to cross-pollination. ?*Atherigona* species often visited the inflorescences but not strictly in synchrony with the flowering events. The species does not seem to serve as an effective pollinator.

Two female flies of *Neurochaeta mcalpinei* were collected only once (Table S3). McAlpine (1987) suggested that the members of *Neurochaeta* have a morphology (flattened body shape) and behaviour (running backwards) that appear to be adapted to host plants in the families Araceae, Musaceae, Pandanaceae and Zingiberaceae. These plants shelter the flies in narrow cavities: the axils of bracts, the sheath hollows of petioles and spathe cavities. One female individual belonging to the species *Stenomicra australis* was also collected (Table S3). McAlpine (1987) noted that flies belonging to *Stenomicra* often share the same habitat as *Neurochaeta*. All of these flies may use *Alocasia* plants as preferred hosts, but there is no evidence that they are effective pollinators.

Earwigs (*Chelisoches morio*) were often seen at the bottom of the spathe chambers and appeared to prey on the eggs and larvae of *Colocasiomyia* and ?*Atherigona* species. Terry (1905) reported the predatory habits of *C. morio* on leafhoppers; this earwig is omnivorous but seems to prefer an insect-based diet (Tenbrink & Hara 2006). Kamimura (2001) observed the nymphs of another earwig species, *Forficula hiromasai* Nishikawa, 1970 (Dermaptera: Forficulidae), on inflorescences of *Arisaema serratum* and *A. thunbergii* (Araceae) and found pollen grains of these plants in the nymph guts. Thus, earwigs may depend on aroid hosts for food (pollen and prey) and habitat (spathe chamber) throughout their life cycle. From our observations, however, there is no indication that earwigs contribute to cross-pollination.

Rove beetles (Coleoptera: Staphylinidae) also visited the inflorescences (Table S3). They were often seen at the inflorescences of *Alocasia*, *Colocasia* and particularly, *Schismatoglottis* in the family Araceae (K. T. Takano and M. J. Toda, unpublished data), presumably targeting eggs and larvae of *Colocasiomyia* or other insects breeding at the site. In this study, the number of rove beetles was very small and they did not seem to function as pollinators.

### Pollination by *Colocasiomyia* flies

The inflorescences bagged with a fine mesh produced almost no fruits (Fig. 4). Ivancic *et al.* (2005) concluded that *A. macrorrhizos* is predominantly self-incompatible. Our bagging experiment with a coarse mesh suggested that the main pollinators could only be small insects that can pass through 2-mm grids. From our observations of the insect visitors and the two bagging experiments, we conclude that the *Colocasiomyia* flies (Sp. 1 and Sp. 2) were the main and possibly only effective pollinators for *A. macrorrhizos* at the study site.

### Reproductive traits of *Colocasiomyia* species

Usually, a pistilicolous and a stamenicolous species of the *Colocasiomyia cristata* group are found within the same inflorescence of *Colocasia* or *Alocasia* but show different traits in niche choice, morphology and life history (Okada 1986). Our results contradicted this pattern because both species reproduced in the female zone and showed only slight differences.

#### Niche choice for oviposition

Both Sp. 1 and Sp. 2 exhibited the pistilicolous habit of oviposition in the female zone (Table S2), and subsequent development until adult eclosion occurred within the infructescence (Table S4). This is the first reported observation of two pistilicolous *Colocasiomyia* species coexisting within a single inflorescence and infructescence. However, some level of niche segregation was still observed because Sp. 2 adults congregated mainly in the upper part of the spathe chamber while those of Sp. 1 gathered in the lower part (Table S3).

#### Morphological characteristics

Stamenicolous *Colocasiomyia* species generally have narrower ovipositors, which are presumably an adaptation for laying eggs in the narrower spaces between stamens. Pistilicolous species have wider ovipositors and lay their eggs in the wider spaces between pistils. The ovipositor was wide (pistilicolous type) in both species, although Sp. 2 had a longer ovipositor compared with Sp. 1 (Takenaka 2006). The following traits of Sp. 2 suggest a stamenicolous tendency: a longer ovipositor, the congregation of adults at the upper part of the spathe chamber and the distinct second peak at the intermediate region of the inflorescence in the egg distribution. The differences between the species may reflect microniche differentiation through reaction to larval food resources or against predators and parasitoid wasps.

#### Life history from egg to eclosion

The life-history traits of both *Colocasiomyia* species reported here strongly indicate intimate adaptation to their host plant. Immatures of stamenicolous *Colocasiomyia* generally leave the inflorescence with decaying tissue when the upper spadix and spathe wither and fall, or ‘pop out’ of the spadix to pupate on the ground (a larval behaviour commonly observed in a number of species of Diptera) (Yafuso 1993), whereas pistilicolous species spend the whole period from egg to eclosion on their host plants. For Sp. 1 and Sp. 2, the time from oviposition to eclosion was estimated to be <62 days in the shortest example and more than 89 days in the longest example. These periods are remarkably long when compared with those of other *Colocasiomyia* species. The respective periods under laboratory conditions are approximately 18 and 30 days for *C. stamenicola* and *C. pistilicola* (Carson & Okada 1980), and 2 and 3 weeks for *C. alocasiae* and *C. xenalocasiae* (Yafuso 1999). In the field, *C. alocasiae* and *C*. *xenalocasiae* seem to require more time (K. T. Takano, M. J. Toda and M. Yafuso, unpublished data), but the periods are still considerably shorter than those reported here. A hole often develops at the apex of *Alocasia* infructescences before dehiscence, because the top part of the spindle of the spadix decays as the infructescence ripens. In contrast, infructescences of *A. macrorrhizos* are completely sealed (Fig. 1T) until dehiscence (Fig. 1U), because the spindle of the spadix remains. Sp. 1 and Sp. 2 may find it difficult to exit before the infructescences dehisce, possibly resulting in the protracted egg-to-eclosion period. Thus, the floral life history of the hosts may explain the variation in the length of the egg-to-eclosion period of *Colocasiomyia* species.

In both species, the protracted egg-to-eclosion period was largely due to the relatively long developmental periods in the third instar and pupa as well as the prolonged residence in the puparium, after metamorphosis to the adult form (Appendices 1, 2). These flies seem to adjust their developmental stages to the conditions of their host plant infructescence. The soakage in the infructescences decreases as the fruits ripen, and the decreasing wetness may be the cue for the larvae to pupate. Adults that have completed metamorphosis within puparia then appear to wait for dehiscence of the infructescence, before leaving the puparium and the plant.

Most adult flies emerged at the same time when the host infructescence dehisced. Rapid and simultaneous departure after dehiscence may be necessary because the mature fruit are frequently eaten by animals (possibly birds or squirrels) after dehiscence and sometimes before dehiscence (Fig. 1W). Exposed puparia (Fig. 1V) may also be attractive for other insects such as ants, which are often observed on the plant. Changes in certain physical conditions such as the light intensity or air composition inside the spathe tube may be cues for eclosion. Experimental investigations such as making a hole in the spathe tube before dehiscence might help to reveal such cues for the eclosion of *Colocasiomyia* species.

### Characteristics of this pollination mutualism

The two *Colocasiomyia* species were host-specific pollinators and depended for their reproduction on inflorescence and infructescence of their host plant. In this sense, this pollination system is comparable to obligate pollination mutualisms in which fig–fig wasp and yucca–yucca moth systems are the best-documented examples (Janzen 1979; Wiebes 1979; Powell 1992; Pellmyr *et al.* 1996). In typical obligate pollination mutualisms, plants have a dilemma: to sacrifice ovules or developing seeds in return for pollination. In contrast, larvae of the *Colocasiomyia* species do not damage fruits, therefore the host plant does not have a dilemma.

Moreover, no adults of the two *Colocasiomyia* species were caught by intensive net sweeping either around the host plants or at other adjacent sites. This suggests that both fly species spend most of their lifetime within the inflorescences of the single host species, except for brief periods of adult migration. It also suggests that *Alocasia macrorrhizos* provides most of necessary resources for its pollinators to survive: food for immatures and adults, place for reproduction (mating and oviposition sites) and shelter for eggs, larvae and adults. Year-round availability of inflorescences may secure continuous reproduction of the flies. However, many of Araceae host plants of other *Colocasiomyia* flies have limited flowering seasons. How *Colocasiomyia* flies survive when host flowers are not available is unknown.

More than 70 *Alocasia* species, about 10 *Colocasia* species and about seven *Steudnera* species are distributed in the Oriental and Papuan regions (Mayo *et al.* 1997). Given the high host specificity of the *Colocasiomyia cristata* group (Sultana *et al.* 2006), there seem to be many undiscovered pollination mutualisms between Araceae plants and *Colocasiomyia* flies. Comparative studies of these presumed pollination systems would shed more light on the evolution of the highly intimate pollination mutualisms (Takano *et al.* 2011).

## Notes

**Table S1.** Diagnostic morphologicalcharacteristics of immatures of *Colocasiomyia* and?*Atherigona* species collected from *Alocasiamacrorrhizos* inflorescences and infructescences in Kota Kinabalu, Sabah, Borneo, Malaysia. **Table S2.** The numbers of individuals in eachdevelopmental stage found in different parts of each inflorescence(stage II and III) or young infructescence (stage IV) of*Alocasia macrorrhizos* in Kota Kinabalu. **Table S3.** The numbers of adult*Colocasiomyia* flies and other insects collected from eachstage-II (female phase) inflorescence of *Alocasiamacrorrhizos* at Kota Kinabalu. **Table S4.** The numbers of *Colocasiomyia*individuals at different developmental stages on eachinfructescences (stages V–VI) of *Alocasia macrorrhizos* at Kota Kinabalu.
