# Supplementary material for: Pollination mutualism between Alocasia macrorrhizos (Araceae) and two taxonomically undescribed Colocasiomyia species (Diptera: Drosophilidae) in Sabah, Borneo
Source: Plant Biol (Stuttg). 2012 Jan 30;14(4):555–64. doi: 10.1111/j.1438-8677.2011.00541.x (PMC3588145; doi:10.1111/j.1438-8677.2011.00541.x)
Supplement: Supplementary file 1 [file plb0014-0555-sd1.doc]

Supplemental File 1. Diagnostic morphological characteristics of immatures of *Colocasiomyia* and ?*Atherigona* species collected from *Alocasia macrorrhizos* inflorescences and infructescences in Kota Kinabalu, Sabah, Borneo, Malaysia.

Immature stage	Species	Description
Egg	Sp. 1	Larger than Sp. 2; relatively straight (gently curved in Sp. 2) along the axis, gently convex (not convex and narrower) ventrally. Surface mat (glossy) white; terminal disk spherical (tabular) with a short filament. Hatching earlier than Sp. 2, from the date of oviposition to the next day.
	Sp. 2	Smallest among the three species; hatching a half to one day later than Sp. 1.
	?*Atherigona* sp.	Biggest among the three species; surface with two conspicuous cristae along the axis, mat white; afloat on water and hardly sink; hatching about concurrently with or later than Sp. 2.
First instar larva	Sp. 1	Body more slender than ?*Atherigona* sp., obviously larger than Sp. 2 at hatching. Caudal abdominal segments elongated, with posterior spiracles with long divergent stalks; spicules on body surface stouter and hooked more strongly than in Sp.2; anterior spiracle almost retracted into the body. Wiggling in the water.
	Sp. 2	General appearance resembling Sp.1; spicules on body surface slightly weaker; mouth hock thinner but curving more strongly than in Sp.1.
	?*Atherigona* sp.	Somewhat roundish overall; mouth hock stout and short; caudal abdominal segment terminated abruptly without elongation; posterior spiracles short and separated each other.
Puparium	Sp. 1	Generally larger Sp. 2; puparial surface glossy and darker (mat and pale in Sp. 2). Anterior spiracles short, but the apical end of the stalk rather distinctive (ambiguous) and the spiracles looking ternate often (not often). Relative length of circular opening of puparium compared to puparial size relatively larger (smaller). Divergent stalks of posterior spiracles as long as or longer (shorter) than one third of that of caudal abdominal segments.
	Sp. 2	Spicules on surface weaker and sparser than in Sp.1.
	?*Atherigona* sp.	Much larger than those of *Colocasiomyia* puparia.

Supplemental File 2. The numbers of individuals in each developmental stage found in different parts of each inflorescence (stage II and III) or young infructescence (stage IV) of *Alocasia macrorrhizos* in Kota Kinabalu.

Flowering / fruiting stage			II				III	Sub total *[2]	IV	
Host ramet No.			201	234	241	242	223		227	227
Inflorescence / infructescence No.			5	8	11	9	9		14	13
Recoded number of days from anthesis			0-1	0-1	0-1	0-1	1-2		6	9
Sp. 1	Section in a inflorescence or infructescence*[1]	4					<u>5</u>	5		
		5					10 , <u>6</u>	16		
		6	5 , <u>1</u>		10		1 , <u>24</u>	41		
		7	<u>1</u>	1	2	<u>5</u>	1 , <u>25</u>	35	<u><u>4</u></u>	
		8	<u>1</u>	9	9 , <u>10</u>	6 , <u>5</u>	<u>30</u>	70	<u><u>12</u></u>	<u><u>2</u></u>
		9	4 , <u>1</u>	19	38 , <u>15</u>	29 , <u>13</u>	2 , <u>30</u>	151	<u><u>77</u></u>	<u><u>25</u></u>
Sp. 2	Section in a inflorescence or infructescence*[1]	4					1 , <u>2</u>	3		
		5	3	5			1 , <u>2</u>	11		
		6	26	2	16	25	<u>1</u>	70		<u><u>1</u></u>
		7	3	2 , <u>5</u>	10	2	1 , <u>2</u>	25	<u><u>5</u></u>	<u><u>1</u></u>
		8		2 , <u>8</u>	6 , <u>1</u>	7	<u>4</u>	28	<u><u>5</u></u>	<u><u>11</u></u>
		9	22	3 , <u><u>11</u></u>	28 , <u>1</u>	21	<u>10</u>	96	<u><u>12</u></u>	<u><u>27</u></u>
?*Atherigona* sp.	Section in a inflorescence or infructescence*[1]	1								<u><u>20</u></u> , (2)
		2		1			7 , <u>2</u>	10		<u><u>1</u></u> , (1)
		3					5 , <u>2</u>	7		<u><u>3</u></u> , (3)
		4								<u><u>1</u></u>
		5		1				1	<u><u>9</u></u>	
		6	2		6			8		
		7								
		8								
		9								

Notes: *n* , eggs; <u>*n*</u> , first instar larvae; <u><u>*n*</u></u> , second and third instar larvae; and (*n*), pupae. *[1] The numbered sections are shown in Fig. 3. *[2] Significantly different between the *Colocasiomyia* species by chi-squared test ($\chi^2 = 407.5$, $df = 8$, $P < 0.0001$).

Supplemental File 3. The numbers of adult *Colocasiomyia* flies and other insects collected from each stage-II (female phase) inflorescence of *Alocasia macrorrhizos* at Kota Kinabalu.

Host ramet No.		205		202		201		234		241		242		227		308		224	
Inflorescence No.		6		2		5		8		11		9		15		5		13	
Part in a inflorescence*[1]		U	L	U	L	U	L	U	L	U	L	U	L	U	L	U	L	U	L
Sp. 1	♀	2	26	2	8	0	22	2	50	19	49	1	46	50	62	2	16	2	66
	♂	3	34	0	3	0	19	1	48	21	70	0	34	34	69	1	24	0	78
Sp. 2	♀	24	0	4	2	13	2	9	1	18	4	5	6	23	12	7	1	25	0
	♂	28	7	11	5	13	2	17	2	33	2	5	3	14	16	11	3	53	8
Difference in species composition*[2]		***		**		***		***		***		***		*		***		***	
Difference in sex ratio*[2]	sp.1	n.s.		n.s.		n.s.		n.s.		n.s.		n.s.		n.s.		n.s.		n.s.	
	sp.2	*		n.s.		n.s.		n.s.		n.s.		n.s.		n.s.		n.s.		n.s.	
Neurochaeta mcalpinei (Diptera: Neurochaetidae)	♀					2													
Stenomicra australis (Diptera: Periscelididae)	♀					1													
?*Atherigona* sp. (Diptera: Muscidae)										1									
Trigona fuscobalteata (Hymenoptera: Apidae)								1											
Parasitoid wasp (Hymenoptera)											2				2				
Staphylinidae species (Coleoptera)										1									
Collembola species								1											

Notes: *[1] U = upper part, L = lower part (Fig. 3) . *[2] Difference in the number of each *Colocasiomyia* species in the upper and the lower parts of each inflorescence by Fisher's exact tests. * = $P < 0.05$, ** = $P < 0.01$, *** = $P < 0.0001$, n.s. = not significant.

Supplemental File 4. The numbers of *Colocasiomyia* individuals at different developmental stages on each infructescences (stages V-VI) of *Alocasia macrorrhizos* at Kota Kinabalu.

	Fruiting stage	V														VI					
	Host ramet No.	223	237	222	241	225	230	228	236	229	231	221	222	227	232	224	223	222	225	226	221
	Infructescence No.	11*[1]	10*[1]	6	6	5	3	3	1	2	2	4	4	3	1	3	1	1	2	1	1
	Recoded (*) or estimated (= P_i) number of days from spathal opening	16*	16*	47.5	47.8	60.2	64.2	64.2	64.2	73.5	75.6	79.6	81.9	85.9	89.0	62.3	73.7	74.4	75.9	77.0	90.1
Sp. 1	Third instar larva	1	10	77																	
	Puparium with undeveloped adult body			1	16	54	81	53		17											
	Puparium with developed adult body (parasited*)				33 (2*)				9	17	16	21	40	74 (3*)	36			1			
	Empty puparium after eclosion								3		2	1		5		21	27	47	22	63	21
	Dead pupa																			9	
	Dead adult failed in eclosion																			10	
Sp. 2	Third instar larva	20	11	20																	
	Puparium with undeveloped adult body				5	32	73	17		16		2		1					1		
	Puparium with developed adult body (parasited*)				2 (14*)			(12*)	17		14	23	23	69	1 (2*)			2	1		
	Empty puparium after eclosion (parasited*)							(1*)	4		4			1		43	22	90	39	16	24

Note: *[1] Used for coarse-meshed bagging experiment.
